# Studies on nanoprotein vaccine alleviating symptoms of mice allergic to rFel d 1

**DOI:** 10.3389/fimmu.2025.1524929

**Published:** 2025-05-26

**Authors:** Yanqi Mai, Xiaozheng Sun, Xiaoxuan Liu, Huricha Chen, Xinglan Liang, Ying Huang, Xiaojuan Wu, Shuangshuang Wei, Dayong Wang, Yechun Pei

**Affiliations:** ^1^ School of Life and Health Sciences, Hainan Province Key Laboratory of One Health, Collaborative Innovation Center of One Health, Hainan University, Haikou, Hainan, China; ^2^ School of Tropical Agriculture and Forestry (School of Agricultural and Rural Affairs, School of Rural Revitalization), Hainan University, Haikou, Hainan, China; ^3^ Hainan International One Health Institute, Hainan University, Haikou, Hainan, China; ^4^ Laboratory of Biopharmaceuticals and Molecular Pharmacology, School of Pharmaceutical Sciences, Hainan University, Haikou, Hainan, China

**Keywords:** rFel d 1 antigen, asthma, vaccine, layered double hydroxides, T cell

## Abstract

**Introduction:**

Globally, cat allergens are a common cause of allergic rhinitis and asthma. Fel d 1 is the primary allergen among cat allergens and can induce a broad range of allergies through airborne transmission.

**Methods:**

In our study, we constructed layered double hydroxide (LDH) nanoparticles loaded with PADRE-rFel d 1, aiming to address allergies triggered by Fel d 1. We utilized a mouse model sensitized with purified rFel d 1 and then immunized them subcutaneously with LDH nanoparticles loaded with PADRE-rFel d 1.

**Results:**

Our results indicated that this nanoparticle vaccine effectively restored the balance of Th1/Th2 and Th17/ Treg cells, which led to a reduction in inflammatory cell infiltration, mitigated local and systemic stress responses induced by rFel d 1, decreased airway hyperresponsiveness, and lowered serum IgE levels.

**Discussion:**

Consequently, the LDH loaded with PADRE-rFel d 1 vaccine shows promise as an effective treatment for cat allergies.

## Introduction

The domestic cats (*Felis domesticus*), as one of the most popular pets, are a rich source of allergens in the environment. Cat allergy is IgE-mediated type I hypersensitivity reaction that impact approximately 20% of individuals worldwide (1–3). The clinically symptoms of cat allergy range from mild rhinitis to life-threatening asthmatic responses (4–6). In the WHO/IUIS allergen nomenclature, a total of eight cat allergens have been identified and named Fel d 1 through Fel d 8. The Felis domesticus allergen 1 (Fel d 1), which is the major allergen of domestic cats predominantly found in the saliva, sebaceous glands, skin, and hair of cats (7), can elicit IgE responses of approximately 90% of individuals who are allergic to cats (8).

Allergic asthma is a chronic inflammatory airway disease characterized by infiltration of inflammatory cells, airway hyperresponsiveness (AHR), and impaired lung function (9–11). Currently, common treatments for allergic asthma include avoiding contact with allergens, pharmacotherapy, and allergen-specific immunotherapy (AIT). However, each method has its drawbacks. Fel d 1 is widely disseminated in the environment by binding to small particles in the air, making it extremely difficult to avoid the allergen (12). Pharmacological interventions, such as intravenous corticosteroids and anticholinergic agents, can only temporarily relieve allergic symptoms, but fail to address the root cause of the disease. Allergen-specific immunotherapy (AIT) is currently the only treatment modality that can modify the natural course of allergic diseases through immune regulatory mechanisms (13). Its therapeutic mechanism mainly involves the long-term administration of minute amounts of allergens through sublingual or subcutaneous routes. The objective is to shift the immune response from the disease-promoting Th2 cells to the non-pathogenic Th1 cells and/or to induce the formation of regulatory T cells (Tregs), which help in modulating the immune system towards a more balanced state, causing a deviation from the pathogenic Th2 towards the non-pathogenic Th1 and/or regulatory T cell (Treg) responses and increasing the production of blocking antibody IgG (14, 15). However, AIT encounters some challenges, including the long duration of treatment, loew patient adherence, and the serious side effects (16).

Therefore, there is a need for a safer, more effective, and cost-effective allergen-specific immunotherapy. Layered double hydroxides (LDHs) are an emerging class of inorganic nanomaterials, belonging to the family of hydrotalcites, which consist of positively charged hexagonal layers and an interlayer structure that can exchange anions (17). Research has indicated that LDHs can activate dendritic cells (18), enhancing immune responses (19). They have been shown to effectively encapsulate antigens and provide a sustained release at the injection site, achieving continuous immune stimulation and reducing the frequency of administration. Additionally, due to their high biocompatibility, stability, biodegradability, and non-toxicity, LDHs hold great promise in the field of drug delivery (20).

In our study, we constructed a murine model sensitized to rFel d 1, aiming to evaluate the efficacy of rFel d 1-loaded LDH nanoparticle-based vaccine in treating allergic mice induced by rFel d 1 and to explore its potential mechanisms of action.

## Methods

### Mice

Adult female BALB/c mice (6–8 weeks) were purchased from SPF (Beijing) Biotechnology Co.,Ltd and maintained in pathogen-free environment with food and water ad libitum. All animal experiments were reviewed and approved by Experimental Animal Committee of Hainan University.

### Cloning, expression and purification of PADRE-rFel d 1

Sequences encoding Fel d 1 chain 1 (GenBank, AAC37318) and chain 2 (GenBank, AAC41616) were retrieved from the NCBI GenBank database and subsequently codon-optimized. The cysteine (Cys) residue at the C-terminus of chain 1 is covalently linked to the valine (Val) residue at the N-terminus of chain 2, resulting in the formation of Fel d 1 (1 + 2) and named rFel d 1. Pan DR T cell epitope (PADRE), a universal T cell epitope, was conjugated to the N-terminus of rFel d 1 via the linker AAY, and this construct was cloned into the expression vector pQE80L. The resultant expression vector was named as pQE80L-PADRE-rFel d 1. The plasmid pQE80L-PADRE-rFel d 1 was transformed into *Escherichia coli* strain BL21 (DE3). Expression of pQE80L-PADRE-rFel d 1 was induced at 37°C with 1 mM IPTG. After 6 h, the bacterial cells were collected after centrifugation (12,000 rpm, 4°C, 30 min), and then resuspended in native lysis buffer (50 mM NaH_2_PO4, 300 mM NaCl and 10 mM imidazol, pH=8.0). The cells were sonicated on ice using a program with 45% power, 30% temperature, 3 s of operation, and 3 s of rest, for a total duration of 10 min. Collecting the protein precipitate after centrifugation (12,000 rpm, 4°C, 15 min). The precipitate was washed sequentially by resuspension in Wash Buffer I (50 mM Tris-HCl, 1 mM EDTA, 100 mM NaCl, 1% Triton X-100, pH=8.5), Inclusion Body Wash Buffer II (50 mM Tris-HCl, 1 mM EDTA, 100 mM NaCl, 1% Triton X-100, 2 M urea, pH=8.5), and Inclusion Body Wash Buffer III (50 mM Tris-HCl, 1 mM EDTA, 100 mM NaCl, 1% Triton X-100, 2 M guanidine hydrochloride, pH=8.5), with each step incubating for 10 min at each step and centrifugation at 4°C, 12,000 rpm for 15 min. The inclusion bodies were solubilized in Inclusion Body Solubilization Buffer I (50 mM Tris-HCl, 1 mM EDTA, 100 mM NaCl, 10 mM DTT, 2 mM sodium deoxycholate, 8 M urea, pH=8.5) at a ratio of 100 µL per gram of wet cells mass, followed by gentle shaking for 1 h. Nine volumes of Inclusion Body Solubilization Buffer II (50 mM KH_2_PO_4_, 1 mM EDTA, 50 mM NaCl, pH=10.7) were added, and the mixture was gently shaken for at least 30 min. After centrifugation (4°C, 12,000-13,000 rpm, 15 min), the supernatant was collected. Adjust the protein concentration to 0.1-1.0 mg/mL and perform gradient dialysis refolding using refolding buffer I/II/III (50 mM Tris-HCI, 100 mM NaCl, 6 M/4 M/2 M Urea, 1% glycine, 5% glycerol, 0.2% PEG 4000, 1 mM oxidized glutathione, 1 mM reduced glutathione, pH=8.5), the refolded protein was dialyzed in PBS for 12 h to remove urea. The protein was further purified through Ni-NTA column affinity chromatography and dialyzed in PBS for 12 h to remove salt.

### Preparation of LDH+PADRE-rFel d 1 vaccine

LDH was synthesized by co-precipitation method. Specifically, Solution B (40 mL 0.15 M NaOH, Xilong Scientific, China) was placed on a magnetic stirrer and stirred while Solution A (3.3 mL ddH_2_O, 5 mL 0.6 M MgCl_2_•6H_2_O and 1.7 mL 0.6 M AlCl_3_•6H_2_O, SCRC, China) was added dropwise. After the addition was completed, the mixture was continuously stirred for 30 min to ensure thorough mixing. Subsequently, the precipitate was collected after centrifugation (RT, 5000 × g, 5 min) and washed twice with pure water, then resuspended in 40 mL of sterile water and subjected to hydrothermal treatment in a 100°C water bath for 16 h. The final product is a transparent LDH suspension. The prepared LDH nanocarriers were characterized by malvern laser particle size analyzer, x-ray diffractometry (XRD), fourier transform infrared spectroscopy (FTIR) and field emission transmission electron microscope (FE-TEM). Mixing the prepared LDH and PADRE-rFel d 1 in various mass ratios (M_LDH_: M_PADRE-rFel d 1_ = 1:1, 2:1, 3:1, 4:1, 5:1, 6:1, 7:1, 8:1, 9:1, 10:1,11:1, 12:1), then shaked at 1000 rpm for 30 min at RT. Following centrifugation (RT, 5000 × g, 5 min), aspirated the supernatant and assessed the adsorption of LDH and PADRE-rFel d 1 using SDS-PAGE electrophoresis.

### Sensitization and vaccination

**Figure 1:**
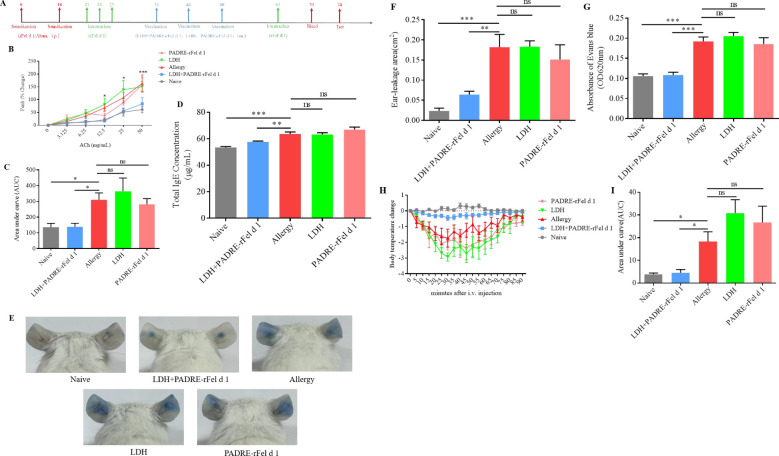
Vaccination with nanoprotein vaccine reduce the allergic symptoms induces by rFel d 1. **(A)** Experimental protocol. **(B)** Airway responsiveness to increasing doses of acetylcholine chloride were determined using whole-body plethysmography and expressed as **(C)** Area under the curve (AUC). **(D)** Levels total IgE in serum. **(E)** Representative pictures of skin active cutaneous anaphylaxis. **(F)** The area of dye leakage analysis by imagej. **(G)** Analysis of Evans Blue absorbance by spectrophotometry (620 nm). **(H)** Change in body temperature after i.v. with rFel d 1. **(I)** The area under the body temperature variation curve. Results are expressed as mean ± SEM of 6 mice per group. One-way ANOVA: ^*^P < 0.05, ^**^P < 0.01, ^***^P < 0.001 different from allergic group; ns, not significantly different from allergic group.

The construction of the therapeutic model for allergic asthma in mice is shown in **Figure 1A**. Specifically, female BALB/c mice, after one week of acclimatization, were randomly divided into five groups (n=6 per group): Naïve group, Allergic group, LDH+PADRE-rFel d 1 group, LDH group and PADRE-rFel d 1 group. Except for the Naïve group, all mice in the other four groups were administered intraperitoneal injections of 100 μ L of allergen rFel d 1 at a concentration of 1 mg/mL (containing 5 mg of aluminum hydroxide adjuvant)on day 0 and day 7. Subsequently, intra-tracheal challenges were performed with 50 μL of rFel d 1 at a concentration of 1 mg/mL on days 21, 23 and 25. Vaccines were administered via multi-point injections at the cervical subcutaneous region and bilateral hind limb muscles on days 32, 46, and 60. Mice in the Sensitized group received 60 μL PBS subcutaneously at the neck and 20 μL PBS intramuscularly in each hind limb. Mice in the LDH+PADRE-rFel d 1 treatment group were immunized with 60 μL subcutaneously at the neck and 20 μL intramuscularly per hind limb of LDH+PADRE-rFel d 1 formulation (800 μg LDH+100 μg protein). Other vaccine groups received an equivalent mass and volume of their respective vaccines via the same routes. A final 50 μL intratracheal challenge of rFel d 1 (1 mg/mL) was administered on day 60. Airway hyperresponsiveness (AHR) was assessed 72 h post-challenge. On day 67, blood was collected from the eye socket to measure total IgE levels in serum. Pathological evaluations were conducted 48 h after blood sampling.

### Airway responsiveness

The airway hyperresponsiveness (AHR) of mice in different treatment groups was measured using whole-body plethysmograph (FinePointe WBP). Specifically, before administering different concentrations of acetylcholine chloride (PBS, 3.125 mg/mL, 6.25 mg/mL, 25 mg/mL, 50 mg/mL), the mice were first placed in the chamber to adapt for 30 min. The nebulization time for each concentration was 1 min, the recorded reaction time was 4 min, and the recovery time was 2 min. The AHR was assessed by measuring the Penh values in mice exposed to different concentrations of acetylcholinechloride (ACh) and calculating the percentage of peak expiratory flow/inspiratory flow ratio (Penh) values relative to those measured during nebulized PBS exposure, as well as the area under the curve.

### Histology and inflammation score

Blind scoring of pulmonary inflammation using H&E, PAS, and Masson staining, with inflammation scores ranging from 0 to 3. Grade “0” indicates no inflammatory cell infiltration around the bronchi, no goblet cells in the lumen, and normal collagen content. Grade “1” indicates occasional inflammatory cells around the bronchi, with 1–5 goblet cells and mild collagen deposition in the lumen. Grade “2” indicates the presence of 1–5 layers of inflammatory cells around the bronchi, with an increase in the number of goblet cells to 6–20 layers, moderate collagen deposition, and persistent fibrosis of the alveolar septa. Grade “3” indicates the presence of more than 5 layers of inflammatory cell infiltration around the bronchi, with over 20 goblet cells within the bronchial lumen, excessive collagen deposition can lead to alveolar wall damage, alveolar compression, and worsening of pulmonary fibrosis.

### ELISA for serum total IgE and allergen-specific IgG, IgG1, IgG2a levels

The total IgE level in mouse serum was quantified by IgE Elisa Assay Kit Instruction (Nanjing Jiancheng Technology Co., Ltd., China), while the levels of rFel d 1-specific IgG, IgG1, IgG2a were detected by indirect ELISA. In brief, 2 μg of rFel d 1 was coated in a high-adsorption 96-well plate and incubated overnight at 4°C. After protein was adsorbed at the bottom of the wells, the plate was washed 5–10 times with 100 μL/well of PBST, followed by blocking the unbound sites with a blocking solution. After sealing at 37°C for 1 h, the plates were washed another 5–10 times with PBST. Serum was used as the primary antibody (diluted 1:250), HRP-conjugated Rabbit anti-mouse IgG (Sangon, China), HRP-conjugated Rabbit anti-mouse IgG1 (Thermo Fisher, USA), HRP-conjugated Rabbit anti-mouse IgG2a (Thermo Fisher, USA) as the secondary antibody (diluted 1:2000) respectively. Upon completion of the antibody incubation, the EL-TMB colorimetric kit (Sangon, China) was used for color development for 30 min, followed by measuring the absorbance at 450 nm.

### Cytokine gene expressions in mouse lungs

IL-5, IL-13, GATA3, RORγt, IFN-γ, T-bet, TGF-β mRNAs in the mice lungs were determined by quantitative real-time PCR and house-keeping-β-actin gene mRNA for RNA normalization, the PCR primers as shown in **Supplementary Table S1**.

### Acute systemic anaphylaxis

For the induction of anaphylaxis, mice were challenged i.v. with 30 μg of rFel d 1/100 μL PBS. Temperature was measured immediately after i.v. antigen challenge and recorded the body-temperature changes of mice from 0 to 90 min after i.v. allergen.

### Ear prick tests

After i.v. injection 200 μL of 0.5% Evans Blue dye for 30 min, 23G puncture needles were used to puncture the center of the left and right ears of mice, and 20 μL of allergen (500 μg/mL) was dropped at the puncture site. After one hour of reaction, the mice were euthanized and the leakage of dye from the ears was observed. For quantification, the ears were cut and weighed, then immerse them in 400 μL of formamide and place them in a 63°C water bath to extract the dye for 32 h. The ears were withdrawn and 100 μL of the extracted contents present in each test tube was pipetted into a 96-well plate. Set two replicates for each sample and measure the absorbance at 620 nm.

### Flow cytometry

After extracting and purifying a single-cell suspension from the spleen, we took three samples from each specimen, each containing 1×10^6^ cells, to analysis of the number of Tregs, Th17, Th1, and Th2 cells. The quantification of Treg cells was achieved through immunostaining with anti-CD4-FITC (Biolegend, USA), anti-CD25-APC (Biolegend, USA), and anti-Foxp3-PE (Biolegend, USA) mAbs. Th17 cells detection were completed by immunostaining with anti-CD4-FITC and anti-IL17A-APC mAbs. For the determination of Th1 and Th2 cells numbers, we also employed immunostaining, utilizing anti-CD4-FITC, anti- IL4-APC, and anti- IFN-γ-PE mAbs.

### Statistical analyses

Statistical analyses were performed using GraphpadPrism. All data were analyzed using one-way ANOVA, followed by *post-hoc* comparisons using the Least Significant Difference (LSD) test and independent t-tests. Values are expressed as mean ± SEM. Statistical significance was defined at a p-value < 0.05 (^*^P < 0.05; ^**^P < 0.01; ^***^P < 0.001).

## Results

### Vaccine preparation

#### Characterization of LDH

**Figure 2:**
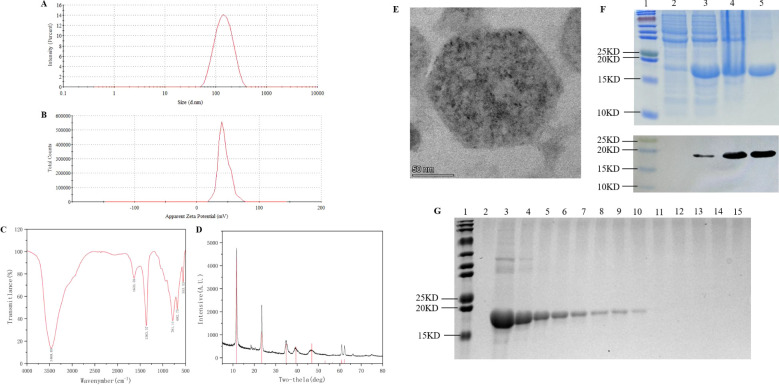
Preparation and determination of nanoprotein vaccine. **(A)** LDH hydrodynamic detection. **(B)** Detection of surface charge on LDH. **(C)** FTIR spectra of LDH. **(D)** XRD patterns of LDH. **(E)** TEM images of LDH. **(F)** SDS-PAGE and immunoblot analysed the prokaryotic expression and purification of PADRE-rFel d 1, lane 1: multicolor prestained protein ladderk (cat. No. WJ106, epizyme, China), lane 2: uninduced *E. coli*, lane3: IPTG-induced *E. coli*, lane4: precipitate after ultrasonic disruption of the cell body post-induction, lane 5: rFel d 1 after purification. **(G)** Adsorption test of LDH with PADRE-rFel d 1. lane 1: multicolor prestained protein ladderk (cat. No. WJ106, epizyme, China), lane 2: LDH, lane 3: PADRE-rFel d 1, lane 4 to lane 15: LDH was loaded with PADRE-rFel d 1 in mass ratios of 1:1, 2:1, 3:1, 4:1, 5:1, 6:1, 7:1, 8:1, 9:1, 10:1,11:1, 12:1, respectively.

LDH was synthesized by co-precipitation method and characterized through laser particle size analyzer, X-ray diffractometer (XRD), Fourier transform infrared spectroscopy (FTIR) and field emission transmission electron microscope (FE-TEM). The particle size of LDH measured by the laser particle size analyzer was 109.4 nm (**Figure 2A**), the potential was 44.2 mV (**Figure 2B**), the polymer dispersion index (PDI) was 0.181, indicating a relatively uniform dispersion. The X-ray diffraction results showed five distinct peaks corresponding to the standard LDH structure: (003), (006), (009), (110), and (113) (**Figure 2C**). Fourier transform infrared spectroscopy was used to analyze the prepared LDH nanoparticles, and the stretching vibration of functional groups was observed at 3469.69 cm^-1^, 1633.59 cm^-1^, 1363.57 cm^-1^, 781.11 cm^-1^, 682.75 cm^-1^ and 553.53 cm^-1^ (**Figure 2D**). The morphology of the LDH prepared in this experiment was observed by FE-TEM, and a clear regular hexagon was observed at a resolution of 50 nm (**Figure 2E**). The above results showed that we have successfully synthesized LDH nanoparticles, which can be used in subsequent experiments.

#### Expression and purification of PADRE-rFel d 1

The prokaryotic expression vector pQE-80L-PADRE-rFel d 1 was transformed into *Escherichia coli* BL21 (DE3) and induced at 37°C for 6 h with 1 mM IPTG. SDS-PAGE electrophoresis and Western Blot analysis showed that compared with the strain without induction (lane 2, **Figure 2F**), the target protein was highly expressed in the host strain (lane 3, **Figure 2F**). Notably, the protein was found in the form of inclusion bodies (lane 4, **Figure 2F**). After purification, a relatively single target protein was obtained (lane 5, **Figure 2F**).

#### Preparation of nanoprotein vaccine

Mixing LDH and PADRE-rFel d 1 at various mass ratios and shaked for 30 min, centrifuged and collected the supernatant for SDS-PAGE electrophoresis (**Figure 2G**). When the mass ratio of LDH to PADRE-rFel d 1 is 8:1, the protein is completely adsorbed by LDH (lane 11, **Figure 2G**), so the mass ratio was selected as the optimal adsorption ratio for the preparation of the vaccine in subsequent studies.

### Nanoprotein vaccine can alleviate rFel d 1-induced allergic symptoms

In order to evaluate whether the PADRE-rFel d 1-loaded LDH nanoparticle-based vaccine could alleviate allergic airway inflammation in rFel d 1-sensitized mice, the sensitized mice were randomly divided into four groups. Seven days after the final allergen challenge, the mice were immunized three times at two-week intervals respectively with LDH+PADRE-rFel d 1, LDH, and PADRE-rFel d 1. The mice in the Naïve group were that without sensitized or vaccinated (**Figure 1A**).

#### Treatment with nanoprotein vaccine reduces AHR

Airway hyperreactivity(AHR)is an important pathological indicator of allergic asthma. Assessment of AHR showe that, compared with the sensitized group of mice, the AHR of the Naïve group and the LDH+PADRE-rFel d 1 protein vaccine group mice significantly decreased at MCh nebulization concentrations of 12.5 mg/mL and 25 mg/mL, and the AHR was extremely significantly reduced at 50 mg/mL. There was no significant difference when the LDH alone immunization group and the PADRE-rFel d 1 protein vaccine alone immunization group were nebulized with different doses of MCh (**Figure 1B**). The area under the curve of the Penh percentage of the Naïve group and the LDH+PADRE-rFel d 1 group mice was significantly lower than that of the allergic group, and the area under the curve of the Penh percentage of the LDH group and the PADRE-rFel d 1 group mice showed no statistical difference compared with the sensitized group of mice (**Figure 1C**), which indicated LDH+rPADRE-rFel d 1 nanoprotein vaccine can alleviate airway hyperresponsiveness induced by rFel d 1.

#### Nanoprotein vaccine decreases the total IgE levels in serum

ELISA method is used to detect the total IgE levels in serum. We found that the total IgE levels in serum of mice in the sensitized group was significantly higher than that of the mice in the Naïve group and LDH+PADRE-rFel d 1 group. There were no statistically significant differences in IgE levels in the serum among the sensitized group, LDH group, and PADRE-rFel d 1 group of mice (**Figure 1D**).

#### Nanoprotein vaccine inhibits rFel d 1-specific actived local or systemic allergic reactions

To inquire into the effects of vaccination on local or systemic allergic reactions, ear skin prick tests with rFel d 1were performed. we observed that following exposure to the allergen rFel d 1, there was a very obvious dye leakage in the allergy group, LDH group and PADRE-rFel d 1 group, while the dye leakage area was significantly reduced in the naïve group and LDH+PADRE-rFel d 1 group (**Figure 1E**). Statistical analysis of Evans blue leakage area also showed that naïve group and LDH+PADRE-rFel d 1 group was significantly smaller than allergy groups (**Figure 1F**).

The ears, after being harvested and soaked in 400 μL of formamide, were subjected to water bath at 64°C for 24–48 h to extract the dye. The absorbance of evans blue at 620 nm revealed a significant reduction in the naïve and LDH+PADRE-rFel d 1 groups relative to the sensitized group (**Figure 1G**). Similar outcomes were noted in systemic allergic reactions, after i.v. with rFel d 1, the naïve and LDH+PADRE-rFel d 1 groups exhibited minimal temperature fluctuations, whereas the sensitized group, LDH group, and PADRE-rFel d 1 group experienced a precipitous drop in temperature, resulting in a significantly larger area under the curve (**Figures 1H, I**).

### Nanoprotein vaccine regulates inflammatory cells infiltration

**Figure 3:**
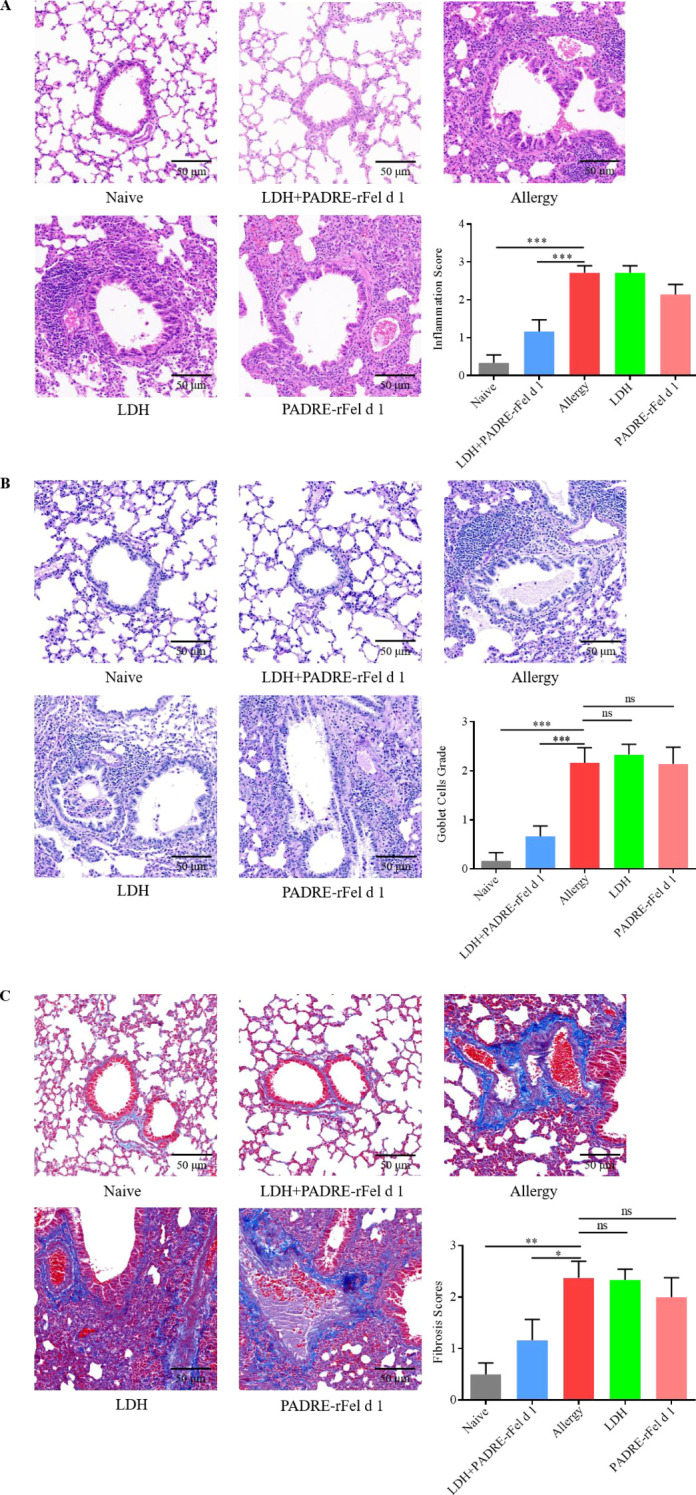
Nanoprotein vaccine immunotherapy prevents rFel d 1-induced lung inflammation. **(A)** Histopathological appearance of mouse lung sections stained with hematoxylin and eosin (H&E) dyes and the peribronchial inflammation score. **(B)** Histopathological appearance of mouse lung sections stained by periodic acid–Schiff (PAS) dye to reveal goblet cells and average goblet cell grades. **(C)** Histopathological appearance of mouse lung sections revealed by Masson’s trichrome staining and average grades of the collagen deposition and fibrotic change. Results are expressed as mean ± SEM of 6 mice per group. One-way ANOVA: ^*^P < 0.05, ^**^P < 0.01, ^***^P < 0.001 different from allergic group; ns, not significantly different from allergic group.

**Figure 4:**
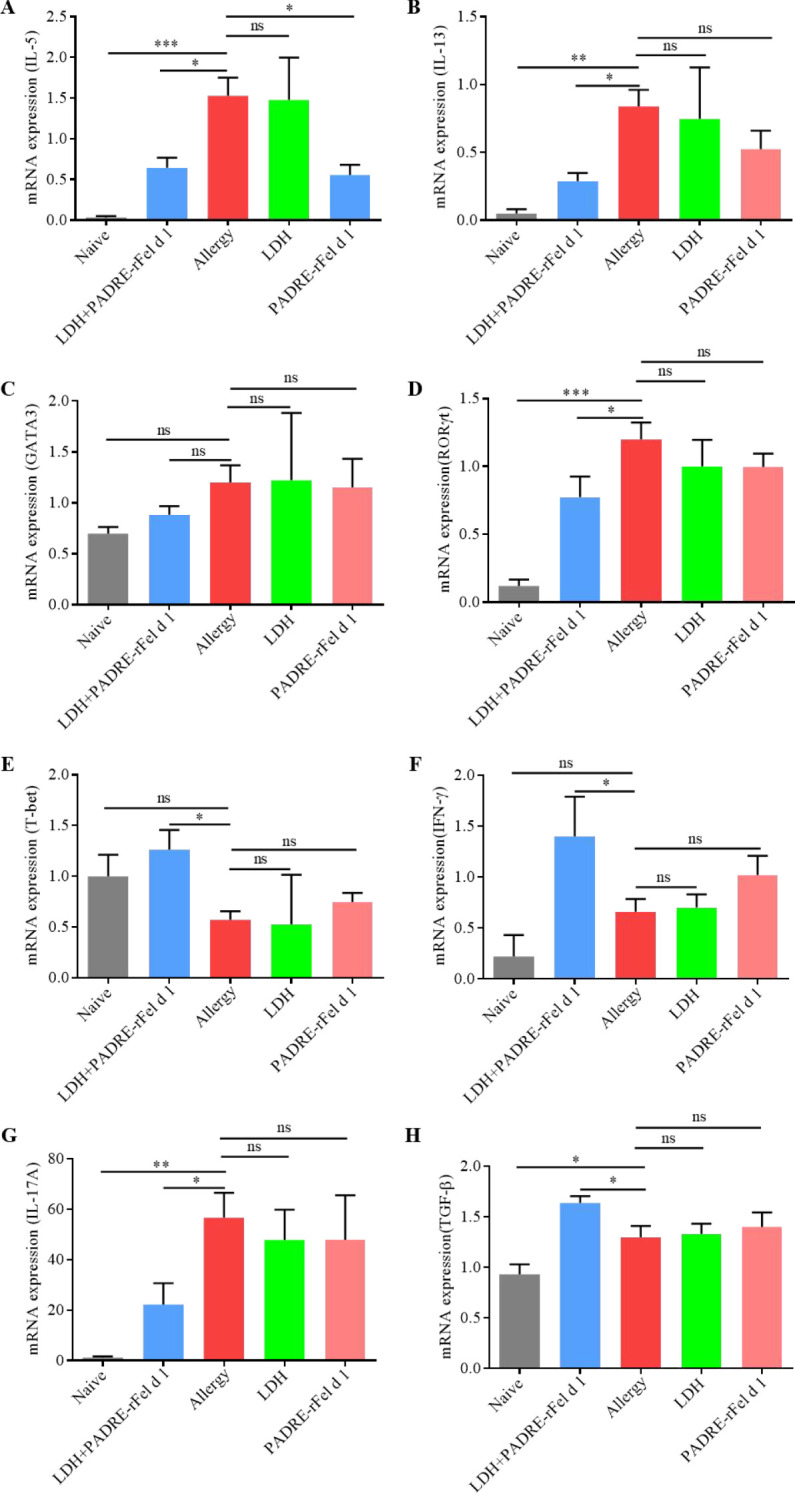
Expression of cytokine genes in the lungs. **(A)** IL-5. **(B)** IL-13. **(C)** GATA3. **(D)** RORγt. **(E)** T-bet. **(F)** IFN-γ. **(G)** IL-17A. **(H)** TGF-β. Results are expressed as mean ± SEM of 4–6 mice per group and are representative of 3 experiments. One-way ANOVA: ^*^P < 0.05, ^**^P < 0.01, ^***^P < 0.001 different from allergic group; ns, not significantly different from allergic group.

Lung inflammatory features of mouse stained by H&E, PAS and Masson are shown in **Figures 1A**, **3A**, **4A**, respectively. The grade of mouse lung inflammatory cells infiltration of naïve group and the LDH+PADRE-rFel d 1 group was significantly lower than that in the sensitized group and other vaccine groups (**Figure 3A**). PAS staining results indicated that the number of goblet cells in the sensitized group, LDH group, and PADRE-rFel d 1 group mice was significantly higher than that in the naïve group and the LDH+PADRE-rFel d 1 group (**Figure 3B**). Similarly, compared with the sensitized group, LDH group, and Protein group mice, the deposition of collagen in the lungs of naïve group and LDH+PADRE-rFel d 1 group mice was significantly downregulated (**Figure 3C**).

### Nanoprotein vaccine modulates the expression of cytokines in lung

As shown in **Figure 4**, compared with the sensitized group of mice, the expression of IL-5 in the lungs of mice treated with LDH+PADRE-rFel d 1 vaccine and PADRE-rFel d 1 vaccine was significantly reduced (**Figure 4A**), and there was a downward trend in the expression of IL-13, GATA3, RORγt, and IL-17A, but only the expression of IL-13 and RORγt in the lungs of mice in the LDH+PADRE-rFel d 1 group was significantly decreased (**Figures 4B–D, G**). The expression of T-bet, IFN-γ, and TGF-β in the lungs of mice treated with LDH+PADRE-rFel d 1 vaccine and PADRE-rFel d 1 vaccine showed a downward trend compared with the sensitized group of mice, among which the expression of T-bet, IFN-γ, and TGF-β in the lungs of mice in the LDH+PADRE-rFel d 1 group was significantly lower than that in the sensitized group of mice (**Figures 4E–H**).

### Nanoprotein vaccine adjusts the balance of Th1/Th2 and Treg/Th17

**Figure 5:**
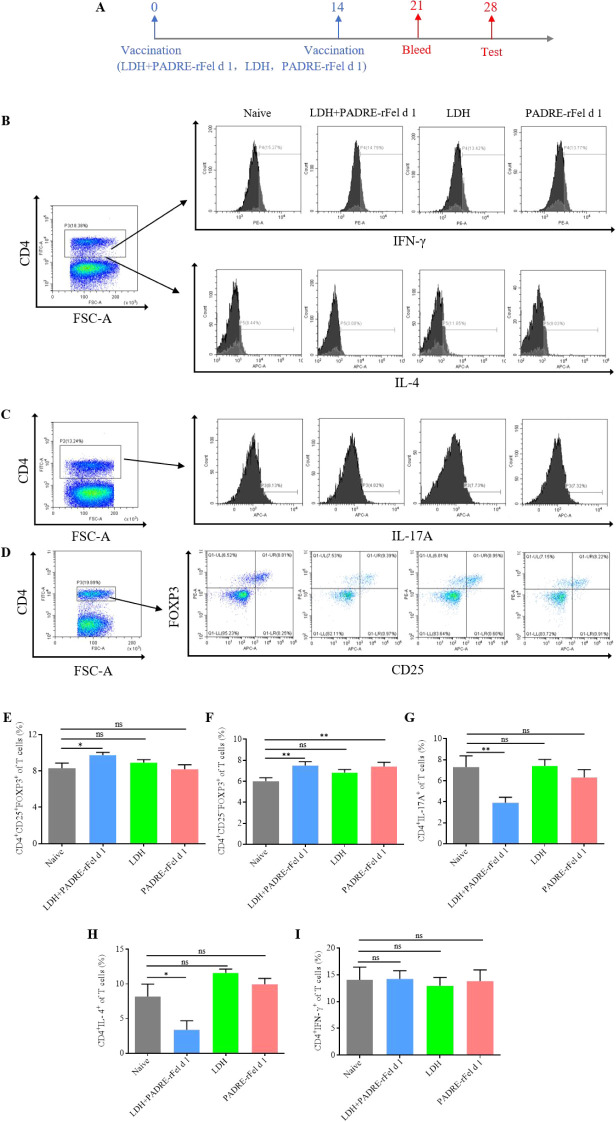
Levels of differential T cells in splenocytes after immunization with rFel d 1-loaded LDH nanoparticle-based nanoprotein vaccine. **(A)** Percentages of CD4^+^IL-4^+^ Th2 cells and CD4^+^ IFN-γ^+^ Th1 cells. **(B)** Percentages of CD4^+^CD25^+^Foxp3^+^ nTreg cells and CD4^+^CD25^-^Foxp3^+^ iTreg cells. **(C)** Percentages of CD4^+^IL-17A^+^ Th17 cells. **(D)** Statistical analysis of percentages of Th2 cells, Th1 cells, nTreg cells, iTreg cells and Th17 cells. Results are expressed as mean ± SEM of 6 mice per group. One-way ANOVA: ^*^P < 0.05, ^**^P < 0.01 different from naive group; ns, not significantly different from naive group.

In order to explore the mechanisms underlying the PADRE-rFel d 1-loaded LDH nanoparticle-based vaccine’s therapeutic effect on rFel d 1-induced allergic responses, vaccinations were given on day 0 and day 14, followed by an assessment of Th1, Th2, Treg, and Th17 cell levels in splenic lymphocytes two weeks post-final immunization (**Figure 5A**). As shown in **Figure 5**, compared to the naïve group, LDH+PADRE-rFel d 1 induced a significant increase in the levels of CD4^+^CD25^-^FOXP3^+^ regulatory T cells and CD4^+^CD25^+^FOXP3^+^ regulatory T cells (**Figures 5D-F**), while significantly downregulating the levels of CD4^+^IL-17A^+^ of T cells (**Figures 5C, G**). PADRE-rFel d 1 significantly suppressed the levels of CD4^+^IL-17A^+^ of T cells (**Figures 5C, G**), but its ability to induce CD4^+^CD25^-^FOXP3^+^ regulatory T cells and CD4^+^CD25^+^FOXP3^+^ regulatory T cells showed no statistical difference compared to the Naïve group (**Figures 5D-F**). Similarly, the same results were observed when examining the levels of CD4^+^IFN-γ^+^ and CD4^+^IL-4^+^ cells. Compared to the naïve group, the levels of CD4^+^IFN-γ^+^ cells in splenic lymphocytes significantly increased, and the levels of CD4^+^IL-4^+^ cells significantly decreased after immunization with LDH+PADRE-rFel d 1. There were no statistical differences in the levels of CD4^+^IFN-γ^+^ and CD4^+^IL-4^+^ cells in mice from the LDH group and the PADRE-rFel d 1 group (**Figures 5B, H, I**).

### Nanoprotein vaccine increases the levels of IgG, IgG1 and IgG2a

**Figure 6:**
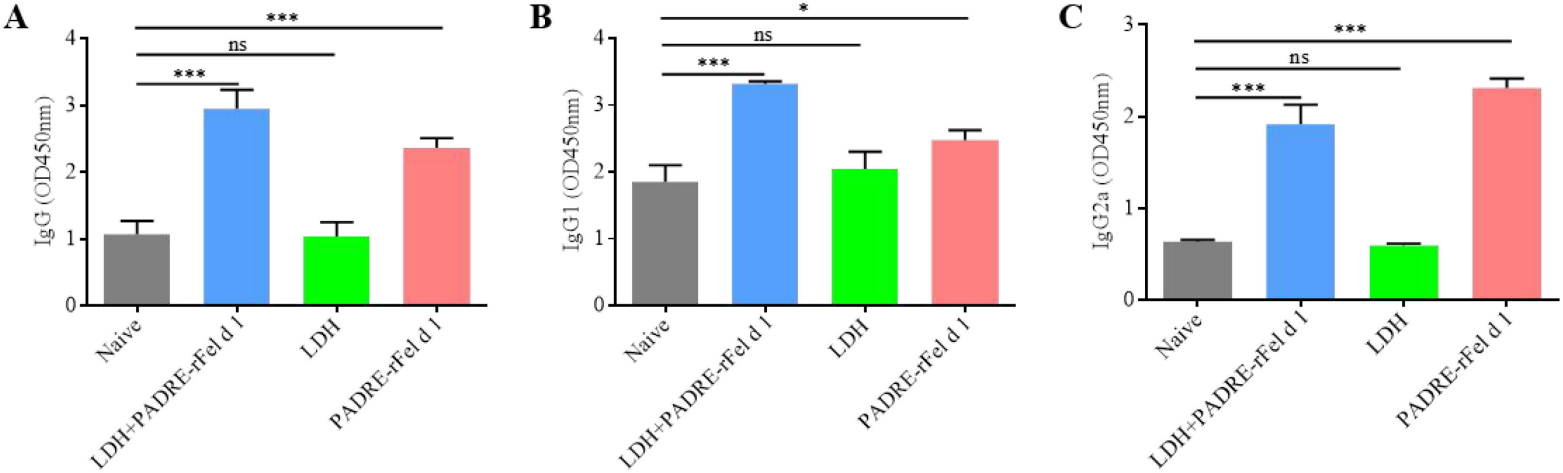
Immunizing mice with nanoprotein vaccine can increase the level of rFel d 1-specific **(A)** IgG, **(B)** IgG1 and **(C)** IgG2a in serum. Results are expressed as mean ± SEM of 6 mice per group and are representative of 3 experiments. One-way ANOVA: ^*^P < 0.05, ^***^P < 0.001 different from allergic group; ns, not significantly different from allergic group.

Two weeks after the second vaccination, the levels of rFel d 1-specific IgG, IgG1 and IgG2a in the mouse serum were measured. As shown in **Figure 6**, compared to the IgG, IgG1 and IgG2a levels in the serum of naive mice, there was no statistically significant difference in the I IgG, IgG1 and IgG2a levels of the LDH group mice, while the levels of IgG, IgG1 and IgG2a in the serum of mice in both the LDH+PADRE-rFel d 1 group and the PADRE-rFel d 1 group were significantly increased (**Figures 6A-C**). Although vaccinated mice showed elevated levels of IgG, IgG1 and IgG2a, further research is needed to determine whether these antibodies exhibit allergen neutralizing or blocking activity, given the complex and potential dual role of IgG subclasses in allergic reactions.

## Discussion

Cat allergy is a global health concern, ranking second in prevalence after dust mites (21, 22). Fel d 1 is the primary allergenic molecule responsible for cat allergies, with more than 90% of cat allergy patients exhibiting Fel d 1-specific IgE in their serum (23, 24). Consequently, immunotherapy targeting Fel d 1 has become a focal point in cat allergy research. In this study, we demonstrated that the developed nano-protein vaccine effectively treats Fel d 1-induced allergic reactions.

PADRE is a non-natural helper T-cell epitope capable of binding to multiple class II MHC molecules, thereby activating CD4^+^ T cells and enhancing the immune response (25, 26). One study indicated that DNA vaccines encoding Ii-PADRE could generate a robust PADRE-specific CD4^+^ T-cell immune response, thereby increasing vaccine efficacy (27). Furthermore, PADRE can form conjugates with specific B-cell epitopes, inducing high-titer IgG antibody responses (28). Research by Parvin Zamani et al. showed that vaccination with a Lip-P5-integrated PADRE-MPL formulation significantly induced IFN-γ production, increased CD8^+^ T-cell numbers, and improved survival rates (29). In our experiment, we optimized the Fel d 1 sequence and linked a universal T-cell epitope (PADRE) to its N-terminus via the AAY linker to enhance the immunogenicity of the protein vaccine. The AAY linker helps maintain the natural conformation and independence of the epitopes, thereby improving the vaccine’s immunogenicity, which is a commonly used linking strategy in multi-epitope vaccine construction (30–32). Hossein Tarrahimofrad and colleagues also found that using the AAY linker enhanced immune responses in the design of a multi-epitope vaccine against influenza A H7N9 (33). Layered double hydroxides (LDHs) are inorganic materials with a layered structure that exhibit significant potential in the biomedical field, particularly as immunoadjuvants due to their unique physicochemical properties and biocompatibility (34, 35). Studies have shown that well-dispersed LDHs can induce stronger cytotoxic T lymphocyte (CTL) responses and significantly inhibit tumor growth (36). Li et al. found that LDH activates dendritic cells through pathways that upregulate CCR7 expression, enhancing dendritic cell migration toward CCL21. Moreover, LDH increases NF-κB expression in the nucleus and promotes IκBα degradation (18). The pH sensitivity of LDH allows for the targeted release of drugs in acidic microenvironments (37), and its biodegradability facilitates the slow release of drugs or antigens *in vivo*, thereby prolonging immune stimulation duration and effectiveness (38). This study employed LDH nanoparticles as an immunoadjuvant, incorporating rFel d 1 into the interlayer of LDH through ion exchange, resulting in the creation of the nano-protein vaccine LDH+PADRE-rFel d 1.

Patients with cat allergies secrete large amounts of allergen-specific IgE, which subsequently binds to high-affinity IgE receptors on the surface of mast cells and basophils, sensitizing these cells. Upon re-exposure to the same allergen, the allergen can bind to IgE on mast cells or basophils, triggering degranulation (39, 40). Pei et al. demonstrated that co-immunization with DNA and protein vaccines significantly reduced serum Fel d 1-specific IgE levels in mice compared to sensitized controls, with stress response assays indicating remarkable therapeutic efficacy (41). A study investigating the potential of TLR9 agonists adsorbed to alum adjuvants in preventing asthma-like reactions induced by tropical mite extracts showed that CpG could inhibit locally or systemically activated allergic responses (42). As highlighted by Khodoun regarding the methodological challenge of IgG/IgE cross-reactivity, positive skin prick test outcomes may result from either IgE antibodies or high-titer IgG antibodies (43). In this study, we specifically addressed this technical ambiguity by employing IgG/Fcγ receptor-pre-adsorbed anti-IgE monoclonal antibodies in our ELISA protocol to biochemically discriminate between IgE-mediated type I hypersensitivity and IgG-mediated type III hypersensitivity. This method has been validated by Khodoun et al. to eliminate cross reactivity. The results showed that, mice treated with LDH+PADRE-rFel d 1 also exhibited a significant reduction in serum total IgE levels and demonstrated notable therapeutic effects in both local and systemic allergic responses. However, it is noteworthy that serum total IgE levels in mice treated with the LDH+PADRE-rFel d 1 significantly still significantly higher than those in the Naïve group mice. This phenomenon may be attributed to the preparation of PADRE-rFel d1 protein vaccine and rFel d 1 allergen, both of which were expressed in *Escherichia coli* BL21. Since the same preparation was used for sensitisation/desensitisation protocols, potential contaminants present in the protein formulations may have participated in both immunological processes, resulting in research results that may not only reflect the host immune response to the target antigen but also unintended reactivity toward residual contaminants. Degranulation of granule cells leads to inflammatory cell infiltration in lung tissues and a series of pathological changes, including epithelial cell damage, increased mucus secretion, smooth muscle cell hyperplasia and hypertrophy, and extracellular matrix remodeling (44–46). These alterations collectively contribute to airway inflammation and hyperreactivity. Certain biological agents, such as omalizumab, target IgE molecules to reduce mast cell and basophil activation, thereby alleviating airway inflammation and hyperreactivity (47). In contrast, Tezepelumab monoclonal antibodies reduce airway inflammation by inhibiting IL-4 and IL-13 signaling (48). In our experiment, we found that LDH+PADRE-rFel d 1 treatment significantly reduced lung inflammatory cell infiltration, goblet cell numbers, and collagen fibrosis compared to sensitized control mice, with airway hyperreactivity assays further confirming the efficacy of the LDH+PADRE-rFel d 1 nano-protein vaccine.

In type I hypersensitivity reactions, Th1 and Th2 cytokines play crucial roles (49–51). IL-4, a central cytokine of Th2 cells, not only promotes IgE production by B cells but also enhances the activation of mast cells and basophils (52). IL-5 promotes the differentiation and maturation of eosinophils in the bone marrow and drives their migration to tissues (53). IL-13 has similar effects to IL-4, facilitating IgE production by B cells (54) and promoting mucus production by goblet cells, thereby inducing airway hyperreactivity (55). IFN-γ is a primary Th1 cytokine that promotes the differentiation of Th0 cells into Th1 cells while inhibiting Th2 cell activity, consequently reducing IL-4 production (56) and inducing mast cell apoptosis (57). In our study, the cytokine profile revealed that, compared to sensitized mice, those treated with the nano-protein vaccine exhibited significantly decreased mRNA expression levels of IL-4, IL-5, IL-13, GATA3, RORγt, and IL-17A in lung tissue, while expression levels of T-bet, IFN-γ, and TGF-β significantly increased. This suggests that the LDH+PADRE-rFel d 1 vaccine can inhibit Th2 and Th17 immune responses while inducing a Th1 immune response. Consistent with our findings, Natt Tasaniyananda et al. reported that a novel nasal liposome-encapsulated vaccine derived from natural Fel d 1 effectively suppressed Th2 immune responses while favoring Th1 immune responses in the treatment of cat allergen-induced rhinitis (58). Similarly, a study on peptide immunotherapy for cockroach extract-induced allergic reactions observed that the peptide vaccine induced Th1 cytokines while suppressing Th2 cytokines (59). Kim et al. noted that oleanolic acid could mitigate OVA-induced airway inflammation and Th2-mediated allergic asthma by modulating the transcription factors T-bet, GATA-3, RORγT, and Foxp3 (60).

The imbalance between Th1/Th2 and Treg/Th17 cell populations is considered a molecular mechanism underlying allergic diseases (61). Research suggests that modulating the balance of these cell subpopulations can alleviate symptoms of allergic diseases. For instance, one study found that quercetin could improve the Th1/Th2 and Treg/Th17 balance, thus relieving symptoms of allergic rhinitis (62). Liu et al. discovered that Majie cataplasm might enhance airway hyperreactivity and inflammation by regulating Th1/Th2/Treg/Th17 balance (63). In our study, flow cytometry was used to investigate the mechanism of action of the LDH+PADRE-rFel d 1 vaccine. The results indicated an increase in CD4^+^CD25^+^FOXP3^+^ Treg cells and CD4^+^IFN-γ^+^ Th1 cells, alongside a decrease in CD4^+^IL-4^+^ Th2 cells and CD4^+^IL-17A^+^ Th17 cells. This suggests that the LDH+PADRE-rFel d 1 vaccine achieves therapeutic effects by restoring the balance of Th1/Th2 and Treg/Th17 cells. CD4^+^CD25^-^ Treg cells are a type of antigen-specific regulatory T cell, and Youmin Kang et al. found that co-immunization with matched DNA and protein vaccines could induce these cells and exert immunosuppressive effects (64). Several studies indicate that iTreg can increase the secretion of IL-10 and TGF-β, thus suppressing allergic responses (65–67).

One explanation for the therapeutic effect of “blocking” antibodies IgG is that they compete with IgE for the binding of allergens (48), another suggests that the process of IgE-facilitated antigen presentation is suppressed (15, 68). Saarne et al., in their investigation of a low-allergen Fel d1 vaccine for cat allergy, noted a marked elevation in IgG, IgG1, and IgG2a levels in the serum of vaccinated mice (69). Consistent with the results of Saarne et al, following a two-week post-vaccination period, we assessed the levels of IgG, IgG1, and IgG2a in serum. We observed that, in comparison to the naïve group, the mice of LDH+PADRE-Fel d 1 vaccine group displayed a pronounced increase in these immunoglobulin levels. When interpreting these findings, careful consideration must be given to the complex and potential dual role of IgG subclasses in allergic reactions. Although IgG antibodies are typically associated with allergen neutralization, there is evidence to suggest that certain subclasses, particularly IgG1 and IgG2a, may induce allergic reactions in mouse models through FcγRIII and FcγRIV mediated mechanisms, respectively (70–72). In our study, an increase in IgG titers was associated with reduced clinical symptoms, suggesting a net protective effect. However, the exact underlying mechanisms, whether they involve allergen neutralization, FcγR competition, or alternative immune regulatory pathways, still need to be elucidated. Future research should directly evaluate the functional properties of these antibodies, including their ability to block IgE allergen interactions or inhibit degranulation of mast cells.

## Conclusion

This study demonstrates that the LDH+PADRE-rFel d 1 nanoprotein vaccine can alleviate rFel d 1-induced allergic reactions by inducing the production of iTreg and restoring Th1/Th2 and Treg/Th17 balance. While vaccination leads to elevated antibody titers, additional functional validation studies are required to characterize the potential protective efficacy of these antibodies. In summary, Further investigation of this vaccine is warranted, as it provides foundational data and theoretical support for the development of therapeutic vaccines for cat allergies.

## Data Availability

The original contributions presented in the study are included in the article/**Supplementary Material**. Further inquiries can be directed to the corresponding authors.
