# Supplementary material for: Studies on nanoprotein vaccine alleviating symptoms of mice allergic to rFel d 1
Source: Front Immunol. 2025 May 26;16:1524929. doi: 10.3389/fimmu.2025.1524929 (PMC12146297; doi:10.3389/fimmu.2025.1524929)
Supplement: Supplementary file 1 [file DataSheet1.pdf]

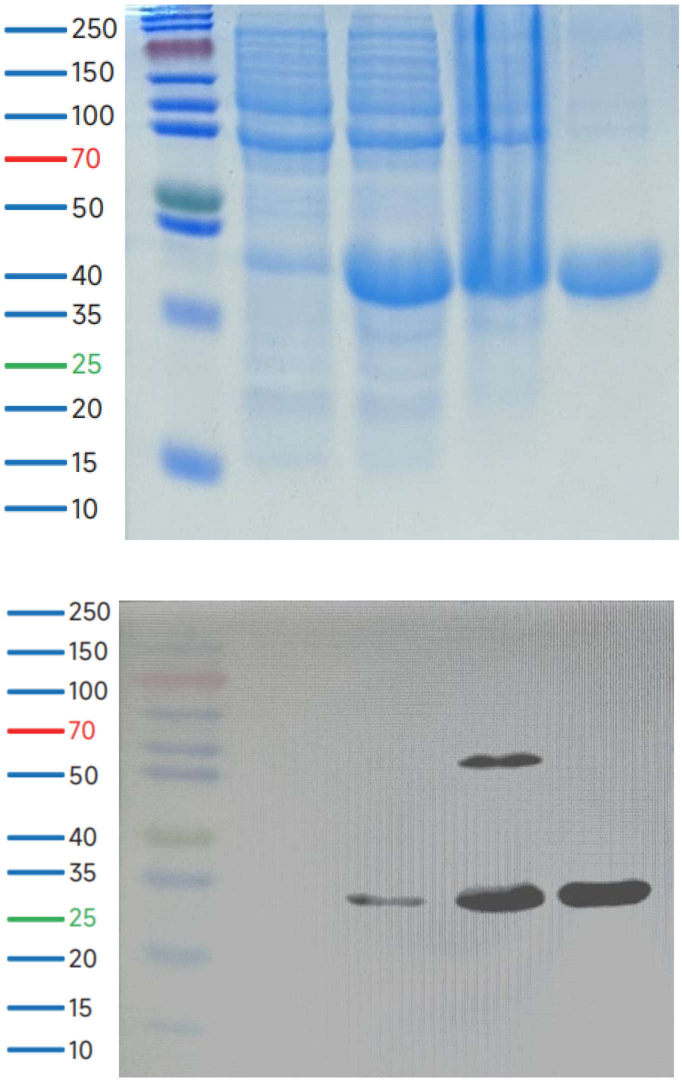

Figure 1F. SDS-PAGE and immunoblot analysed the prokaryotic expression and purification of PADRE-rFel d 1

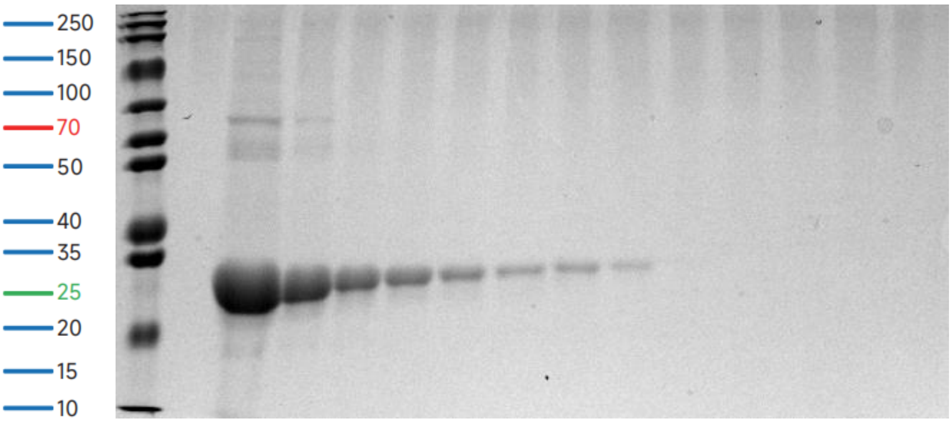

Figure 1G. Adsorption test of LDH with PADRE-rFel d 1.

Supplementary Table 1. Oligonucleotide primers used in qRT-PCR for monitoring cytokine gene expression.

Gene	Primer sequence	Size of PCR product (bp)
IL-5	F: 5′-CCCTCATCCTCTTCGTTGCAT-3′	21
	R: 5′-ATGTGATCCTCCTGCGTCCAT-3′	21
IL-13	F: 5′-GGCAGCAGCTTGAGCACATT-3′	20
	R: 5′-GGCATAGGCAGCAAACCATG-3′	20
GATA3	F: 5′-CAGAACCGGCCCCTTATCA-3′	19
	R: 5′-ACAGTTCGCGCAGGATGTC-3′	19
RORγt	F: 5′-ACAGCCACTGCATTCCCAGTTT-3′	22
	R: 5′-TCTCGGAAGGACTTGCAGACAT-3′	22
T-bet	F: 5′-CCTCTTCTATCCAACCAGTATC-3′	22
	R: 5′-CTCCGCTTCATAACTGTGT-3′	19
IFN-γ	F: 5′-TCAAGTGGCATAGATGTGGAAGAA-3′	24
	R: 5′-TGGCTCTGCAGGATTTTCATG-3′	21
IL-17A	F: 5′-CAGCAGCGATCATCCCTCAAAG-3′	22
	R: 5′-CAGGACCAGGATCTCTTGCTG-3′	21
TGF-β	F: 5′-CACCGGAGAGCCCTGGATA-3′	19
	R: 5′-TGTACAGCTGCCGCACACA-3′	19
β-actin	F: 5′-AGTGTGACGTTGACATCCGTA-3′	21
	R: 5′-GCCAGAGCAGTAATCTCCTTCT-3′	22
